# Multiple Myeloma With Mixed Lytic and Blastic Bone Lesions With Lymphadenopathy: Rare Manifestation of a Common Disease-Case Presentation and Literature Review

**DOI:** 10.4021/wjon440w

**Published:** 2012-04-23

**Authors:** Setu Patolia, Frances Schmidt, Swati Patolia, Neerja Gulati, Perwaiz Muhammad, Dharani Narendra, Danilo Enriquez, Joseph Quist

**Affiliations:** a Interfaith Medical Center, Brooklyn, NY, USA

## Abstract

Multiple myeloma - a neoplastic proliferation of plasma cell is the second most common blood cancer. Multiple myeloma is characterized by neoplastic proliferation of the plasma cells. These cells infiltrate variety of organs. Infiltration by immature neoplastic cells and overproduction of monoclonal immunoglobulin chain is responsible for clinical manifestations of multiple myeloma. The most common clinical presentation of multiple myeloma is an asymptomatic person having anemia and elevated globulin in laboratory testing. Multiple myeloma is diagnosed by triad of > 10% marrow infiltration by plasma cells, serum/urine monoclonal protein and end organ damages. One of the common end organ damage is lytic bone lesions resulting from imbalance between osteolytic and osteoblastic activities. Lymphadenopathy and osteoblastic lesions are rare presentations of multiple myeloma - lymphadenopathy in 1% of cases with IgA subtype and osteoblastic lesions in IgE myeloma and lambda light chains. Osteoblastic multiple myeloma is a distinct entity from POEMS syndrome. IgG myeloma with kappa chain predominance is not described yet with osteoblastic lesions and lymphadenopathy. We present a rare case of IgG myeloma with kappa chain predominance that had both lymphadenopathy and osteoblastic lesions.

## Introduction

Multiple myeloma is neoplastic monoclonal proliferation of plasma cells. Disease is characterized by triad of bone marrow infiltration by plasma cells, lytic bone lesions and presence of M protein in serum/urine. Multiple myeloma usually presents as anemia, renal failure and bone pain. Other common features include fatigue, hypercalcemia and weight loss [1]. Multiple myeloma is rarely associated with lymphadneopathy [2]. Osteoblastic lesions are rarely described in cases of lambda chain predominance multiple myeloma. Osteoblastic lesions are also described in POEMS (Polyneuropathy, Organomegaly, Endocrinopathy, Multiple myeloma and Skin changes) syndrome. Osteoblastic lesions have never been described with IgG myeloma with kappa light chain predominance. With this case report, we present a case of patient admitted with multiple myeloma, diagnosed as IgG with kappa light chain, who had both lymphadenopathy and mixed lytic and sclerotic lesions. We will discuss diagnostic criteria for multiple myeloma and POEMS syndrome and mechanism responsible for osteolytic lesions. We will also discuss the review of the literature for osteoblastic lesions in multiple myeloma.

## Case Report

A 70 year old male without any significant past medical history presented in the emergency department with chief complaints of severe weakness, anorexia and 30 lbs weight loss in last 3 months. He also complained of feeling very tired and shortness of breath. From unrestricted physical activity, his functional capacity had reduced to few blocks and was limited by tiredness. Patient denied any other complaints. Patient was a 100 pack year smoker and ex alcohol abuser. Last physician visit was for hernia repair ten years ago. Patient denied the use of any prescribed or over the counter medication.

**Figure 1:**
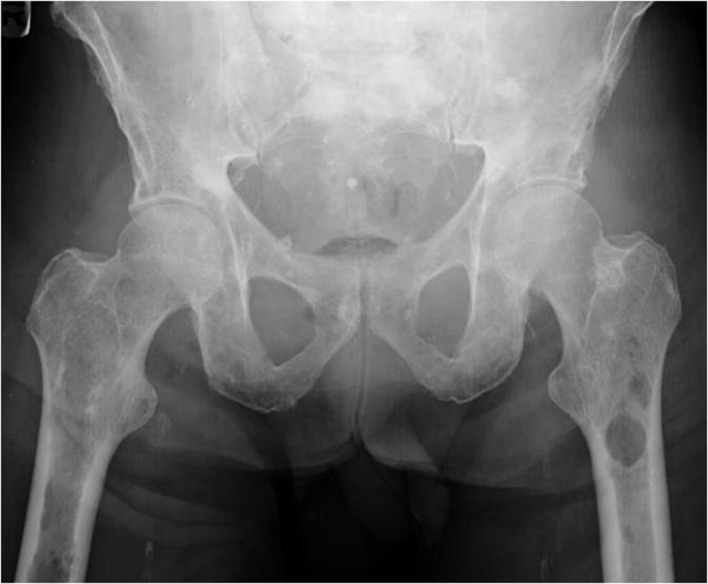
X ray of the pelvis showing multiple osteolytic lesions in the left femur.

**Figure 2:**
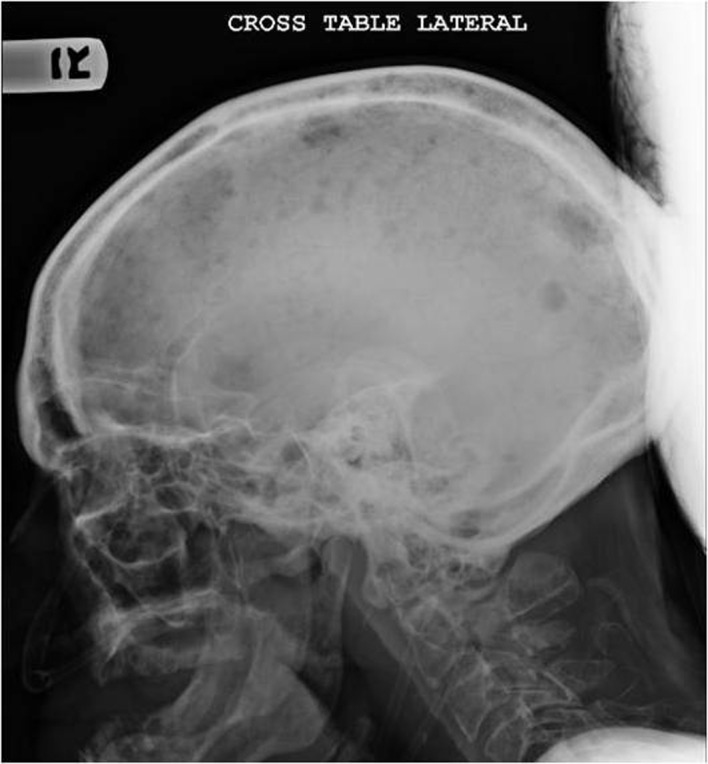
X ray of the skull showing multiple osteolytic lesions in the skull.

On admission patient was afebrile, hypotensive with blood pressure of 97/65, relatively bradycardic with heart rate of 67/minute and tachypneic with respiratory rate of 39/minute. General examination revealed cachectic old male with mild respiratory distress. Physical examination revealed bilateral cervical, axillary and inguinal hard, non matted lymphadenopathy; tachycardia and tachypnea. Physical examination was unremarkable otherwise including sensory and motor examinations. Laboratory investigations revealed leucocytosis with WBC count of 21,000/µL, normocytic normochromic anemia with hemoglobin of 7.6 g/dl. Blood chemistry showed sodium of 121 mmol/l, potassium 6.2 mmol/l, bicarbonate 5 mmol/l, BUN 95 mg/dl, serum creatinine 4.8 mg/dl, albumin of 1.8 g/dl, total protein of 8 g/dl, alkaline phosphates of 154 IU/l and corrected anion gap of 19.5. Blood gas analysis revealed mixed anion and non anion gap metabolic acidosis. Urine toxicology was negative including for alcohol. Urine analysis revealed proteinuria and hemoglobinuria. Other lab tests showed serum osmolarity of 320 mOsm/kg; urine osmolarity of 348 mOsm/kg and fractional excretion of sodium (FeNa) of 6.59. Chest X ray revealed multiple lytic areas in ribs and humerus with fracture of left 9th rib. X ray of the pelvis and x ray of the skull showed multiple lytic lesions (Fig. 1, Fig. 2). Patient was admitted with acute renal failure to rule out multiple myeloma and adrenal insufficiency.

**Figure 3:**
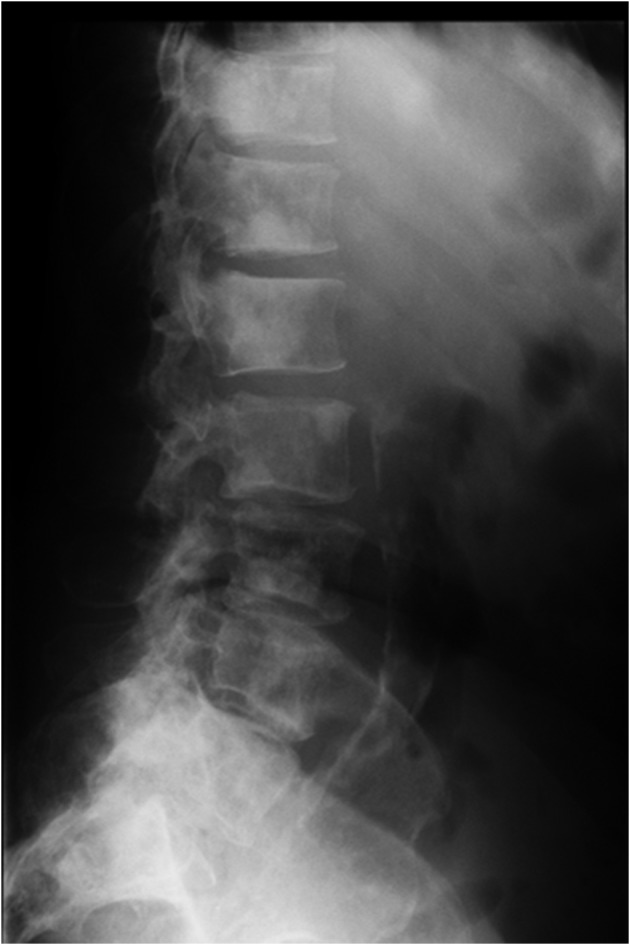
X ray of the thoraco-lumbar vertebrae showing osteoblastic lesions.

**Figure 4:**
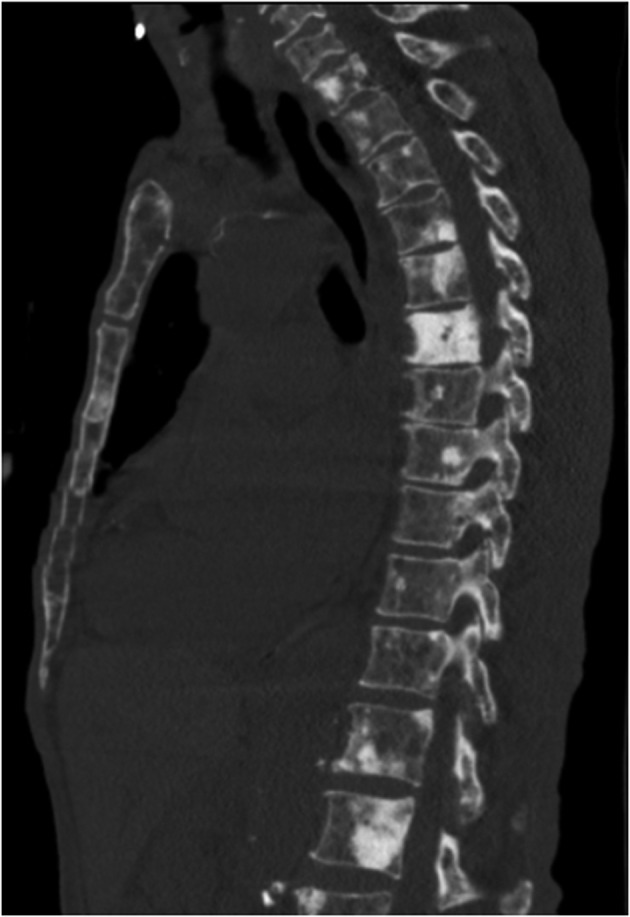
Reconstruction images of the CT chest showing multiple osteoblastic lesions in thoraco-lumbar vertebrae.

**Figure 5:**
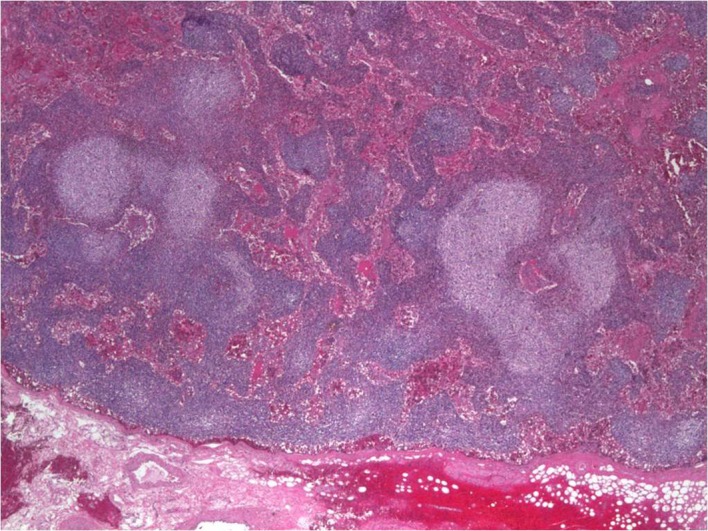
Axillary lymph node biopsy showing reactive follicular hyperplasia with dermatopathic changes.

Serum electrophoresis revealed M spike with elevated IgG monoclonal protein with kappa light specificity. Urine also showed bence-jonce proteinuria with free kappa light chain. Beta 2 microglobulin levels were 32.5 mg/L. Cosyntropin stimulation test revealed primary adrenal insufficiency. Patient was started on IV hydration, dexamethasone and fludrocortisone. Patient’s electrolyte normalized on treatment and renal failure improved. Bone survey revealed lytic and blastic lesions in the vertebrae and other bones (Fig. 3). CT scan of chest also revealed large left 9th rib lesion with extra pleural mass effect. CT chest reconstruction revealed same blastic lesions in thoracolumbar vertebrae (Fig. 4). PSA, CEA and CA 19 - 9 levels were within normal limits and pelvic sonogram did not reveal any prostate nodule. HIV testing was negative and hepatitis testing revealed past infection with hepatitis B. Lymph node biopsy revealed reactive follicular hyperplasia with dermatopathic changes (Fig. 5). No other focus of malignancy was noted on CT chest, abdomen, pelvis and head. Before bone biopsy, patient’s condition deteriorated rapidly and developed DIC, GI bleed and retroperitoneal hematoma which lead to demise of the patient. Based on available data diagnosis of multiple myeloma with IgG kappa chain specificity was made.

## Discussion

Multiple myeloma (MM) is the second most common blood cancer in US and it constitutes 1% of all cancer [3], 5 year survival rate for multiple myeloma is around 40% [4]. MM is characterized by monoclonal proliferation of plasma cells which infiltrates various organs including bone marrow and produces monoclonal immunoglobulin in excess amounts. Etiology of multiple myeloma remains unclear. In studies, a variety of risk factors has been identified and they include - radiation exposure [5]; occupational exposure [6, 7]; hair dyes [8]; chronic antigenic stimulation related to infection or systemic inflammation; viral infections like hepatitis c and HHV [5] and possible role of obesity [5, 9]. None of these risk factors has been proven consistently associated with multiple myeloma. Chromosomal abnormalities are found in 31% of multiple myeloma patients - more than half with non hyper-diploid abnormalities on chromosome 11, 6, 16, 20 and 4. Hyper-diploid abnormalities are found on chromosome 3, 5, 7, 9, 11, 15, 19, 21, 52 - 56 [10, 11].

The neoplastic plasma cells infiltrate bone, bone marrow and other organs. Usual manifestations of multiple myeloma include- osteolytic bone lesions, anemia, hypercalcemia and renal failure. Pleural effusion and diffuse pulmonary involvement is a rare presentation of advanced disease. Diagnosis of MM requires presence of 3 features [12]: 1) More than 10% monoclonal plasma cells in the bone marrow or histological proof of plasmacytoma; 2) Serum or Urine monoclonal protein; 3) End organ damage related to multiple myeloma: lytic lesions in the bone, Hypercalcemia, Anemia or Renal failure.

Lytic bone lesions in multiple myeloma represent uncoupling between osteolytic and osteoblastic activities favoring more to osteolytic activities. Variety of mediators like IL-1, IL-1β, IL-6, sIL-6R, TNF-α, MIP-1α, receptor activator of NF-κB ligand, macrophage inflammatory protein 1 alpha, dickkopf 1, osteoprotegerin and parathyroid hormone–related protein has been implicated for this uncoupling [1, 13-17]. Because of lytic lesions and osteoporosis associated with multiple myeloma, patients have 3 times higher risk of pathological fracture of vertebra and ribs as compared to general population. Risk of fracture is higher in patients with elevated calcium and patients on oral corticosteroids [18].

Lymphadenopathy and blastic bone lesions are rare manifestations of MM. Kyle et al. found that out of 1027 patient diagnosed with multiple myeloma, lymph node enlargement and splenomegaly was present in 1% of the patient, hepatomegaly was present in 4% of the patient. In the same study, lytic lesions were present in 66% of the patient and in 83% of the patient with bone marrow infiltration >10%. Blastic lesions were observed in only 0.5% of the patient [2]. Lymphadenopathy is usually described with IgA myeloma [19]. As compared to MM, lymphadenopathy is found more commonly in Waldenstrom’s macroglobulinemia [17]. Similarly, very few cases of multiple myeloma are reported with diffuse sclerotic and lytic bone lesions. Most of the patients with sclerotic bone lesions described in literature had lambda chain monoclonal gammopathy [20, 21]. A few cases of sclerotic bone lesions with IgE myeloma has also been described in the literature [22]. But sclerotic bone lesions with IgG myeloma with kappa chain specificity have not been described in literature.

Sclerotic lesions are also seen in POEMS syndrome, prostate cancer and patients treated for multiple myeloma. Criteria for diagnosis of POEMS syndrome includes: (1) Monoclonal plasma cell disorder (2) Peripheral neuropathy (3) one of the following: osteosclerotic bone lesions, Castleman’s disease, organomegaly, endocrinopathy (excluding diabetes mellitus or hypothyroidism), edema, typical skin changes and papilloedema. All three criteria must be met to diagnose POEMS syndrome [23]. Pathogenesis of sclerotic lesions in multiple myeloma remains unclear. Martha Q Lacy et al. suggested that possible etiology for sclerotic lesions in the multiple myeloma are either uncoupling of osteoclastic and osteoblastic activity or increased activity of platelet derived growth factors [21].

Our case does not meet criteria for POEMS syndrome - neuropathy which is critical as per Mayo clinic criteria for the diagnosis was absent in our case and our patient’s lymph node biopsy did not reveal Castleman’s disease. Our case has some unique presentations of multiple myeloma. Lymphadenopathy and osteoblastic lesions with IgG kappa chain predominance has never been described in literature with a case of multiple myeloma.
